# Immunohistochemical examination using the pericyte marker myosin 1B in a perivascular myoid tumor of soft tissue with definitive pericytic differentiation

**DOI:** 10.1111/pin.12777

**Published:** 2019-02-21

**Authors:** Shiori Meguro, Sayomi Matsushima, Yasunori Enomoto, Hideya Kawasaki, Isao Kosugi, Takashi Tsuchida, Satoshi Baba, Hidekazu Fukamizu, Yu Yamato, Toshihide Iwashita

**Affiliations:** ^1^ Department of Regenerative and Infectious Pathology Hamamatsu University School of Medicine Shizuoka Japan; ^2^ Department of Diagnostic Pathology Hamamatsu University Hospital Shizuoka Japan; ^3^ Department of Plastic and Reconstructive Surgery Hamamatsu University School of Medicine Shizuoka Japan; ^4^ Department of Orthopediatric Surgery Hamamatsu University School of Medicine Shizuoka Japan

To the Editor:

Herein, we report a soft tissue tumor case arising from a distal extremity that was shown to be composed of tumor cells with definitive pericytic differentiation through immunohistochemical analysis with the pericyte marker myosin 1B (MYO1B).^1^ In our previous study, we identified MYO1B as a new pericyte marker that is expressed in pericytes but not vascular smooth muscle cells (VSMCs). Based on the expression pattern of MYO1B and high molecular weight caldesmon (hCD; a specific marker for SMCs), vascular mural cells were classified into three types of cells, α‐smooth muscle actin (αSMA)‐positive (+)/MYO1B(+)/hCD‐negative (−) pericytes, αSMA(+)MYO1B(−)hCD(+) VSMCs, and αSMA(+)MYO1B(+)hCD(+) vascular mural cells with intermediate features. We then applied this vascular mural cell classification for perivascular myoid tumors (glomus tumor and myopericytoma) and discovered that αSMA(+)MYO1B(+)hCD(−) tumor cells with pericytic features were only found in glomus tumors but not myopericytomas. However, the proportion of αSMA(+)MYO1B(+)hCD(−) tumor cells with pericytic features in 24 glomus tumor cases ranged from 0% to 40%. Furthermore, we have not previously encountered perivascular myoid tumors that were entirely composed of tumor cells with definitive pericytic differentiation.

A 69‐year‐old Japanese male noticed a tumor nodule on his right forearm 10 years ago that has since then gradually increased in size. No specific clinical signs or symptoms of the tumor had been noted. He was admitted to Hamamatsu University Hospital, after which the tumor (located in the subepidermis) was diagnosed as an epidermal cyst and resected. In its gross dimensions, the lesion was a 30 × 20 × 20 mm encapsulated and well‐circumscribed tumor with necrosis at its center (Fig. 1a). Microscopically, the central part of the tumor was necrotic (Fig. S1a). The living oval‐shaped tumor cells with branching smaller vessels were present at the peripheral part of the nodule (Fig. 1b, c). In addition, dilated cavernous spaces were observed. Mitosis was rare, with no more than one cell undergoing mitosis in 10 high‐powered (400×) fields, indicating that this tumor displayed low proliferative ability (Fig. 1c). The original pathological diagnosis was benign perivascular myoid tumor of uncertain differentiation. However, according to the literature on perivascular myoid tumors, the tumor was shown to be histologically similar to glomangiopericytoma arising from a body extremity. Glomangiopericytoma was first described almost two decades ago as well‐circumscribed soft tissue tumors arising from extremities that exhibited features of perivascular myoid tumors with hemangiopericytoma‐like patterns, which was based solely on histological findings without immunohistochemical analysis.^2^ In addition, it was reported that hemorrhaging commonly accompanied these tumors and that one case out of nine showed massive central coagulative necrosis, as seen in our study case. Thus, the histological characteristics of the tumor appeared similar with that of the glomangiopericytoma described in the Granter *et al*.^2^ study.

**Figure 1:**
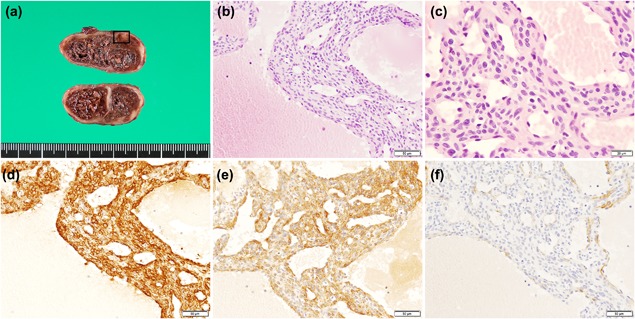
Histology and immunohistochemical staining. (**a**) A gross view of the tumor. The excised surface of the tumor appears well‐circumscribed and with central necrosis. (**b**) Hematoxylin and eosin (H&E)‐stained image of the rectangle in (**a**). The living oval‐shaped tumor cells with glomangiopericytoma‐like pattern of smaller vessels are present at the peripheral part of the nodule. (**c**) Higher magnified image of (**b**). The tumor cells are oval‐shaped without atypia. Mitotic cells were very rare. Immunohistochemical staining indicate that the tumor cells are positive for (**d**) αSMA and (**e**) MYO1B but negative for (**f**) hCD. Scale bars = 10 mm (**a**), 50 μm (**b**, **d–f**), and 20 μm (**c**).

Immunohistochemical staining demonstrated that the tumor cells were positive for αSMA (Fig. 1d) and MYO1B (Fig. 1e) while negative for hCD (Fig. 1f). Additional immunohistochemical staining revealed that the tumor cells were negative for desmin, CD34, and STAT6 (Fig. S1b–d). β‐catenin expression in the nuclei was not detected, unlike for sinonasal glomangiopericytomas (Fig. S1e). The present tumor was shown to be entirely composed of αSMA(+)MYO1B(+)hCD(−) tumor cells with pericytic features, indicating that the tumor in question was a perivascular myoid tumor with definitive pericytic differentiation. Antibodies used in this study are detailed in the Table S1.

Currently, the term “glomangiopericytoma” has been used for sinonasal glomangiopericytomas, which arise exclusively from the nasal cavity and paranasal sinus.^3^ To the best of our knowledge, only two glomangiopericytoma cases occurring outside the nasal cavity and paranasal sinus have been reported after the Granter *et al*.^2^ paper was published.^4^, ^5^ These two cases were diagnosed as glomangiopericytomas based on histological findings and αSMA expression; however, pericytic features of tumor cells in those two cases could not be demonstrated.

Although we had not encountered glomus tumors such as the present case that were entirely composed of αSMA(+)MYO1B(+)hCD(−) cells, it may be a special glomus tumor in which tumor cells are fully differentiated into pericytes. Therefore, it is necessary to investigate whether glomangiopericytoma‐like tumors arising from a distal extremity should be separated from glomus tumors as a distinct clinicopathological entity.

Ultrastructural analysis can also be employed to examine pericyte characteristics, but the fine structure is not always well preserved in formalin‐fixed, paraffin‐embedded tissues. Indeed, we attempted to perform ultrastructural analysis of the tumor, but the fine structure of the tumor cells was considerably destroyed by formalin fixation. Therefore, immunohistochemical analysis using antibodies against MYO1B in addition to αSMA and hCD can be an alternative method for demonstrating pericytic features of tumor cells.

In conclusion, we identified a soft tissue tumor with definitive pericytic differentiation arising from a distal extremity. By using antibodies against the pericyte marker MYO1B in addition to αSMA and hCD, more soft tissue tumors with definitive pericytic differentiation arising from a distal extremity can be discriminated from other histologically similar soft tissue tumors; moreover, the characteristics of those tumors can be revealed.

## DISCLOSURE STATEMENT

None declared.

## Supporting information

Additional Supporting Information may be found in the online version of this article at the publisher's website.

**Figure S1**. Histology and immunohistochemical staining. (a) H&E‐stained sample of the central necrotic tissue. Immunohistochemical staining indicate that the tumor cells are negative for (b) desmin, (c) CD34, (d) STAT6, and (e) β‐catenin. Scale bars = 200 µm (a) and 50 µm (b–e).

**Table S1**. Antibodies used in this study, and method of antigen retrival.
