# Supplementary figures and images for: Immunohistochemical examination using the pericyte marker myosin 1B in a perivascular myoid tumor of soft tissue with definitive pericytic differentiation
Source: Pathol Int. 2019 Feb 21;69(4):246–8. doi: 10.1111/pin.12777 (PMC6850187; doi:10.1111/pin.12777)

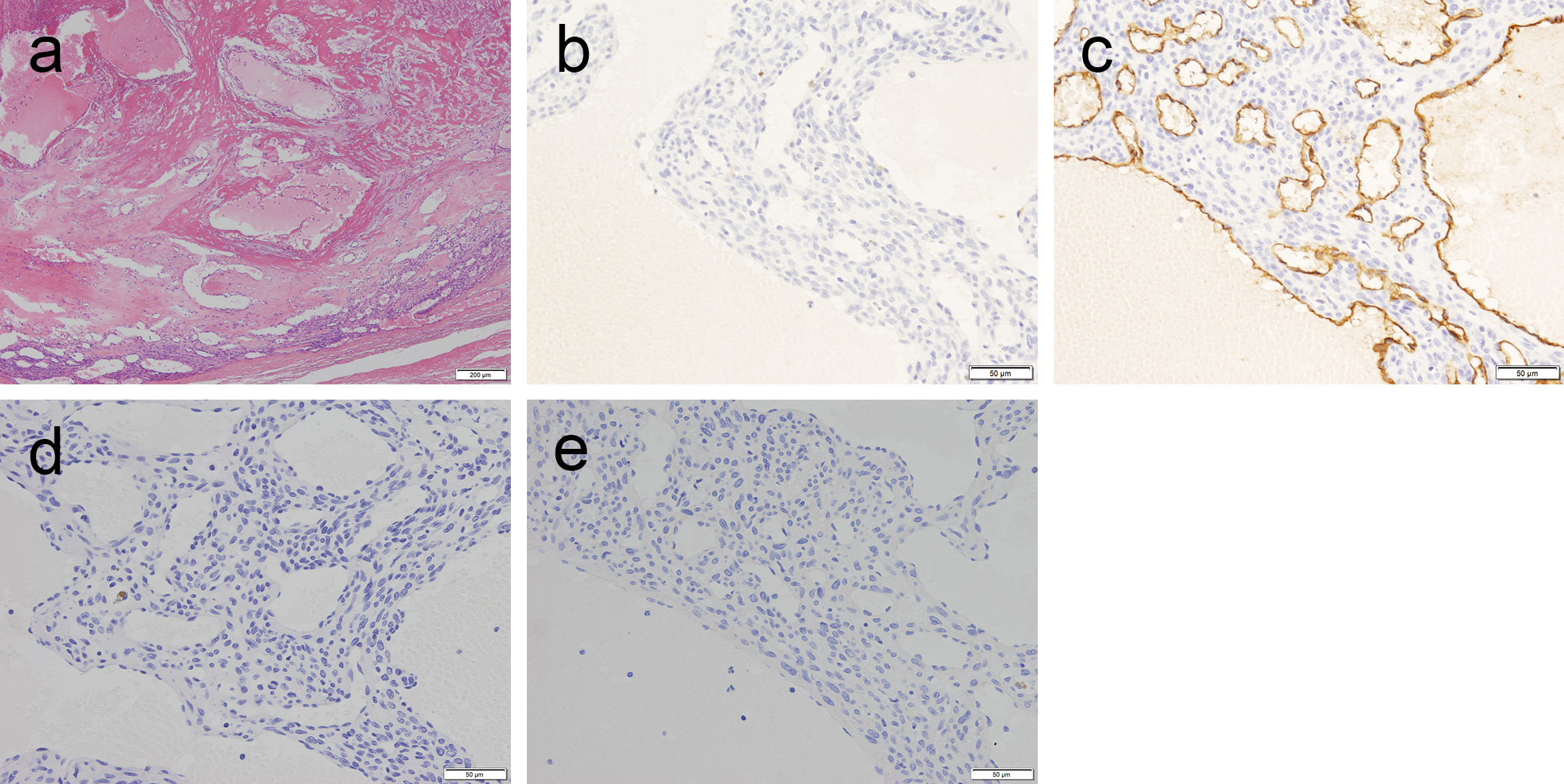

Supplement: Supplementary file 1 — Figure S1. Histology and immunohistochemical staining. (a) H&E‐stained sample of the central necrotic tissue. Immunohistochemical staining indicate that the tumor cells are negative for (b) desmin, (c) CD34, (d) STAT6, and (e) β‐catenin. Scale bars = 200 µm (a) and 50 µm (b–e). [file PIN-69-246-s001.tif]
